# VEGF and FGF2 Improve Revascularization, Survival, and Oocyte Quality of Cryopreserved, Subcutaneously-Transplanted Mouse Ovarian Tissues

**DOI:** 10.3390/ijms17081237

**Published:** 2016-07-30

**Authors:** Sheng-Hsiang Li, Yuh-Ming Hwu, Chung-Hao Lu, Hsiao-Ho Chang, Cheng-En Hsieh, Robert Kuo-Kuang Lee

**Affiliations:** 1 Department of Medical Research, Mackay Memorial Hospital, Tamsui District, New Taipei City 251, Taiwan; lsh@mmh.org.tw (S.-H.L.); hwu4416@yahoo.com.tw (Y.-M.H.); smallriver220@gmail.com (H.-H.C.); 2 Mackay Junior College of Medicine, Nursing, and Management, Beitou District, Taipei City 112, Taiwan; 3 Department of Obstetrics and Gynecology, Mackay Memorial Hospital, Taipei City 104, Taiwan; d95642001@gmail.com (C.-H.L.); shitxn@gmail.com (C.-E.H.); 4 Mackay Medical College, Sanzhi District, New Taipei City 252, Taiwan; 5 Department of Obstetrics and Gynecology, Taipei Medical University, Taipei City 110, Taiwan

**Keywords:** angiogenic factor, ovarian cryopreservation, ovarian transplantation, oocyte quality, fertility reservation

## Abstract

This study was conducted to investigate the effect of the vascular endothelial growth factor (VEGF) and fibroblast growth factor 2 (FGF2) on revascularization, survival, and oocyte quality of cryopreserved, subcutaneously-transplanted mouse ovarian tissue. Autologous subcutaneous transplantation of vitrified-thawed mouse ovarian tissues treated with (experimental group) or without (control group) VEGF and FGF2 was performed. After transplantation to the inguinal region for two or three weeks, graft survival, angiogenesis, follicle development, and oocyte quality were examined after gonadotropin administration. VEGF coupled with FGF2 (VEGF/FGF2) promoted revascularization and significantly increased the survival rate of subcutaneously-transplanted cryopreserved ovarian tissues compared with untreated controls. The two growth factors did not show long-term effects on the ovarian grafts. In contrast to the untreated ovarian grafts, active folliculogenesis was revealed as the number of follicles at various stages and of mature oocytes in antral follicles after gonadotropin administration were remarkably higher in the VEGF/FGF2-treated groups. Although the fertilization rate was similar between the VEGF/FGF2 and control groups, the oocyte quality was much better in the VEGF/FGF2-treated grafts as demonstrated by the higher ratio of blastocyst development. Introducing angiogenic factors, such as VEGF and FGF2, may be a promising strategy to improve revascularization, survival, and oocyte quality of cryopreserved, subcutaneously-transplanted mouse ovarian tissue.

## 1. Introduction

Cryopreservation of ovarian tissue is rapidly evolving and is a promising clinical technique for preserving gonadal reproductive function. It avoids the necessity for ovarian stimulation and stores a substantial amount of ovarian tissue [1,2]. Cryopreservation of ovarian tissue is feasible to restore fertility for young women requiring urgent treatment for cancer and is even the only option to preserve fertility in children with cancer [3].

While cryopreservation and transplantation of the entire ovary takes advantage of immediate restoration of the blood supply by vascular anastomosis [4,5,6], storage of ovarian tissue slices is more practical [2,7]. Previous studies have demonstrated human live birth after orthotopic transplantation of cryopreserved ovarian tissues [8,9,10]. In mammals, several cases of successful reproduction from heterotopic transplantation into the kidney capsule of cryopreserved ovarian tissues also were reported [11,12,13]. Despite the successes in orthotopic and renal capsular transplantation, patients must endure the risk of operation, and ovum retrieval in these locations is very difficult.

In contrast to the aforementioned transplantation, subcutaneous transplantation, such as in the forearm or abdominal wall, is of practical value because it is easily accessible for ovum retrieval [2,14,15] and follicle development is relatively easy to monitor [14,15]. Lee et al. reported the birth of a monkey following in vitro fertilization (IVF) of an oocyte obtained from subcutaneously-transplanted fresh ovarian tissue [14]. Oktay et al. reported that a four-cell human embryo was produced from subcutaneously transplanted cryopreserved ovarian tissue, but no conception occurred [16]; however, spontaneous conceptions and live birth following the subcutaneous transplantation of frozen banked ovarian tissue from a Hodgkin lymphoma survivor were also reported [17]. In mice, subcutaneous grafted ovaries demonstrated poorer graft survival and lower numbers of retrieved oocytes compared with those grafted under the renal capsule or in the bursal cavity [18]. Nevertheless, blastocyst development and live pups from subcutaneous transplantation of cryopreserved ovaries have been reported [19]. Despite some cases of successful reproduction, the technique of subcutaneous transplantation remains premature and needs more investigations.

Follicle loss in the cryopreserved grafts was very common. The key factor is ischemic injury during the early revascularization period of ovarian transplantation [3]. The kidney capsule with ample capillaries provides an ideal grafting site for rapid revascularization. Several previous reports demonstrated that the ovary and renal capsules are rich in angiogenic factors, such as vascular endothelial growth factor (VEGF) and fibroblast growth factor (FGF), which may enhance angiogenesis and promote graft survival [20,21,22,23].

Here, we encapsulated VEGF and FGF2 with vitrified-thawed mouse ovarian tissues and attempted to induce the production of blood vessels in subcutaneously-transplanted cryopreserved mouse ovarian tissue. We evaluated the effect of the two angiogenic factors on revascularization and survival of the ovarian grafts, and subsequent oocyte maturation, fertilization, and embryo development.

## 2. Results

**Figure A1:**
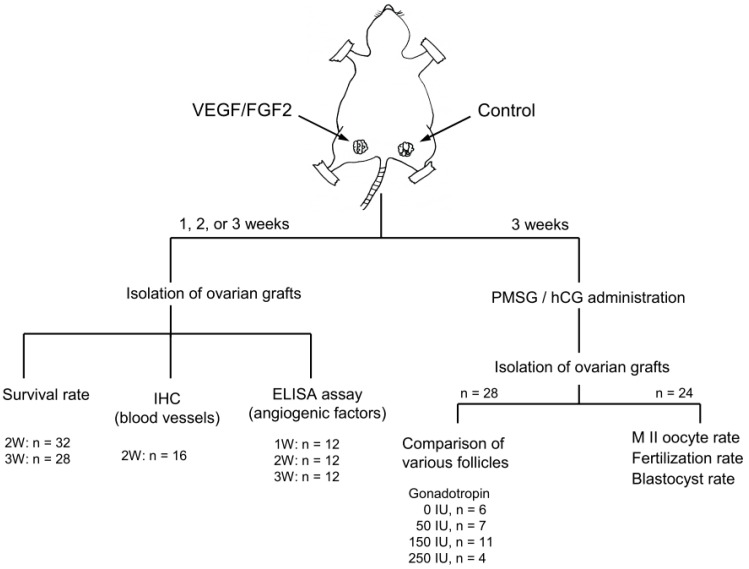
Experimental scheme of this study. The cryopreserved mouse ovarian tissues were autologously transplanted to the subcutaneous site of the inguinal region. The VEGF/FGF2-treated ovarian tissues were implanted into one side of the subcutaneous space, and the ones without treatment of VEGF/FGF2 were inserted into the opposite side of the same mouse. For the assay purpose, ovarian grafts were removed one, two, or three weeks after transplantation as shown on the diagram.

We removed ovarian tissues after subcutaneous transplantation of the VEGF/FGF2-treated ovarian tissues for one, two, or three weeks. Following the experimental scheme in Figure A1, we assessed angiogenesis, survival rate, and oocyte quality of the grafted ovarian tissues.

### 2.1. VEGF Coupled with FGF2 Improved Angiogenesis and Survival of Cryopreserved, Subcutaneously-Transplanted Mouse Ovarian Tissues

**Figure 1:**
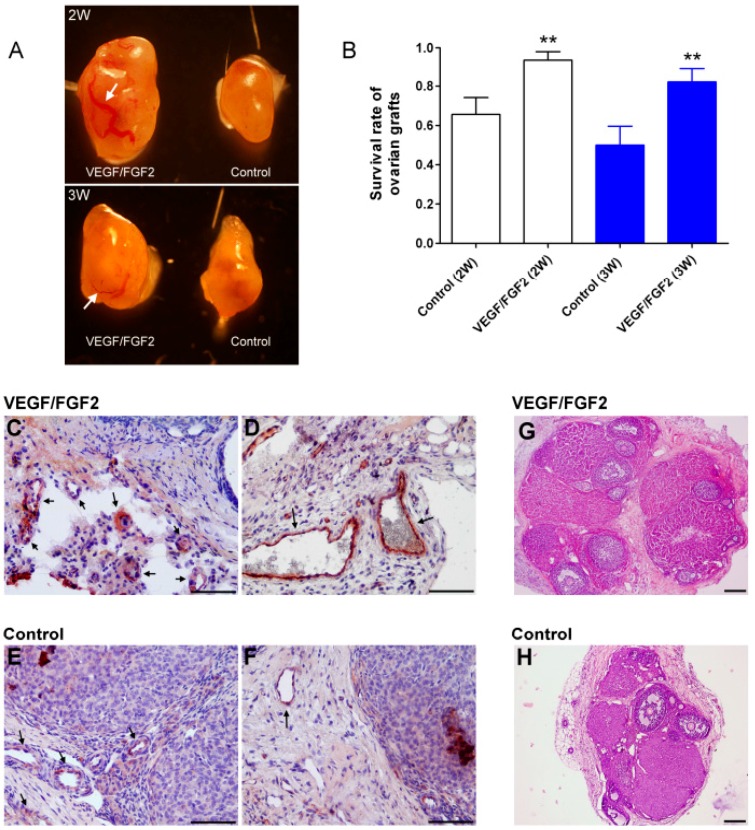
Morphology, survival rate, and blood vessels of the cryopreserved ovarian tissue after transplantation. (**A**) The morphologies of the grafted ovarian tissue. **Left**: VEGF/FGF2-treated tissue; **right**: untreated control tissue. An arrow shows the larger blood vessel; (**B**) The survival rate of the grafted ovarian tissue two and three weeks after transplantation. Survival rate is defined in the text. ** *p* < 0.01 compared with relative control group; (**C**–**F**) Immunohistochemical staining of vessels. The von Willebrand factor (vWF) protein, a marker of the endothelial cells of blood vessels, was stained red-brown (where arrow pointed); (**G**,**H**) Representative photos of H&E-stained ovarian sections show the morphology of ovarian grafts two weeks after transplantation. Scale bars: 100 µm.

After cryopreserved ovarian tissues were transplanted subcutaneously for two or three weeks, the grafted tissues were removed and their appearance evaluated. The VEGF/FGF2-treated ovarian tissue demonstrated a substantially larger size compared with the untreated controls. More blood vessels, even larger vessels, were readily visualized in VEGF/FGF2-treated ovarian tissues, while only microvessels were observed in the control grafts (Figure 1A). Tissue fibrosis occurred in approximately 34% and 50% of control implants as well as 6% and 18% of VEGF/FGF2-treated grafts two and three weeks after transplantation (Table S1). Nevertheless, the tissue survival rate was significantly enhanced under the treatment of VEGF and FGF2. In addition, the grafted tissues two weeks after transplantation seemed to have better survival rates than those at three weeks and no statistical difference was observed (Figure 1B). Histological analysis revealed that the density of small (Figure 1C) and large (Figure 1D) blood vessels was markedly detected in the VEGF/FGF2-treated ovarian tissues, while nearly only small vessels were found in the untreated controls (Figure 1E,F). In addition, more follicles were obviously detected in the VEGF/FGF2-treated ovarian tissues (Figure 1G) compared with the untreated controls (Figure 1H).

### 2.2. Levels of Angiogenic Cytokines in the Ovarian Grafts

**Figure 2:**
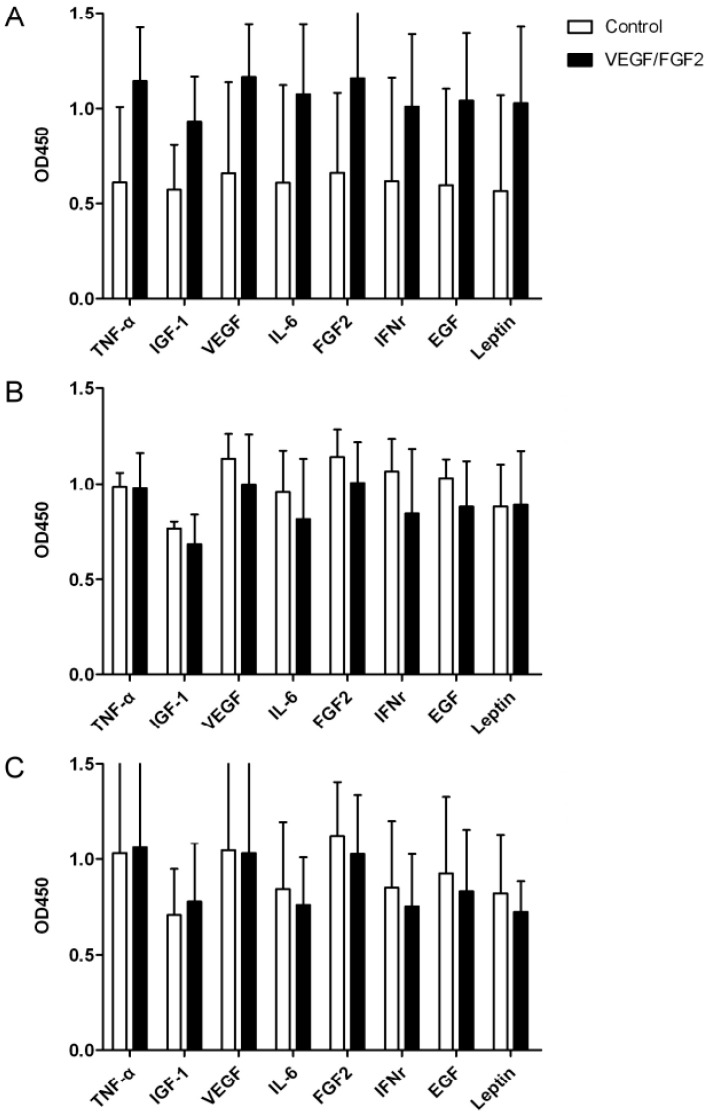
Analysis of protein levels of angiogenic cytokines in the grafted ovarian tissues treated with or without VEGF and FGF2. The grafted ovarian tissues one, two, and three weeks (**A**–**C**, respectively) after transplantation were retrieved. The relative amount of angiogenic cytokines determined by ELISA was represented as bar charts.

We examined the relative levels of angiogenic cytokines, including TNF-α, IGF-1, VEGF, IL-6, FGF2, IFNγ, EGF, and leptin, in the ovarian grafts 1, 2, or 3 weeks after transplantation. All proteins demonstrated higher levels in VEGF/FGF2-treated grafts compared with untreated controls one week after transplantation (Figure 2), though there was no statistical differences between the groups (Figure 2A); however, protein levels were similar two or three weeks after transplantation (Figure 2B,C).

### 2.3. VEGF and FGF2 Improved Oocyte Quality of the Transplanted Ovarian Tissues

Untreated control had a significantly increased number of preantral follicles and an increased trend of antral follicles, despite there was no significant difference, compared with VEGF/FGF2-treated ovarian grafts three weeks after transplantation; however, when different doses of gonadotropins were administered to assess folliculogenesis and oocyte quality of the ovarian grafts, VEGF/FGF2-treated ovarian grafts demonstrated relatively active folliculogenesis compared with the untreated grafts in the administration of various doses of gonadotropins. As the doses of gonadotropins increased, the numbers of primordial, primary, preantral, and antral follicles significantly increased or had an increased trend in VEGF/FGF2-treated groups (Table 1).

**Figure 3:**
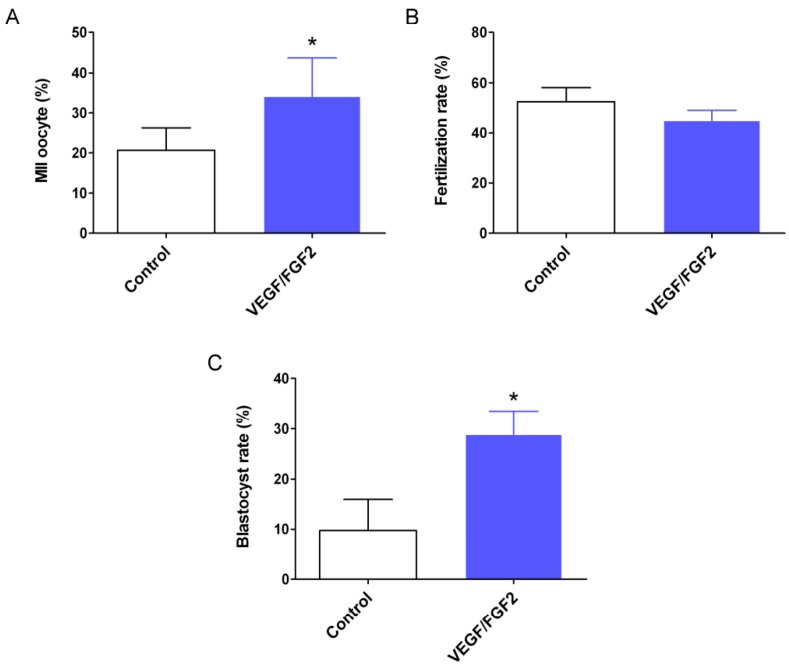
The effect of VEGF and FGF2 on the rate of metaphase II (MII) oocyte, fertilization, and blastocyst development. Mice (*n* = 24) grafted with ovarian tissue three weeks after transplantation were treated with 150 IU gonadotropins, and MII oocytes were collected from the antral follicles for in vitro fertilization (IVF). Fertilization oocytes were cultured to the blastocyst stage. (**A**) The rate of MII oocytes; (**B**) fertilization rate; and (**C**) the rate of blastocyst formation. These results were statistically analyzed using the relative percentage data shown in Table S3. Values are the mean ± SD of six independent experiments (*n* = 4 each). * *p* < 0.05 compared with the control group.

The status of oocytes in the antral follicles of the ovarian grafts is shown in Table 2 and Table S2. The total number of oocytes retrieved from VEGF/FGF2-treated ovarian grafts was higher than that of controls, 191 vs. 162, respectively. The number of matured oocytes was significantly higher in the VEGF/FGF2-treated grafts than that in the controls (Figure 3A). As metaphase II (MII) oocytes were subjected to IVF, the fertilization rate was comparable between the two groups (Figure 3B). However, the zygotes developed to the blastocyst stage were significantly enhanced in the VEGF/FGF2-treated grafts (Figure 3C).

## 3. Discussion

We examined the effect of exogenous angiogenic factors, i.e., VEGF and FGF2, on revascularization, survival, and oocyte quality of cryopreserved, subcutaneously-transplanted murine ovarian tissue. The results revealed that VEGF and FGF2 induced angiogenesis and enhanced the survival of the ovarian grafts and, thus, the oocyte quality was also improved.

Cryopreserved ovarian tissues are subjected to freezing and thawing injury, while grafting to a subcutaneous site causes ischemic injury and also potentially exposes the tissue to altered temperature than normal ovaries [16,24]. Presumably, these injuries impair the quality of the oocyte and subsequent embryogenesis. Due to the lack of vessels under the skin, ischemia occurring during the critical time for revascularization is the primary injury to subcutaneously transplanted ovarian tissue, leading to tissue fibrosis or severe follicle loss [3].

Heterotopic transplantation of the ovarian tissue to the kidney capsules of nude mice led to better follicle development [25,26]. Renal capsules express rich angiogenic factors, such as VEGF and FGF2 and, thus, may enhance graft survival and revascularization [21,22]. To reduce the ischemic injury during subcutaneous transplantation, we encapsulated ovarian tissues with VEGF, FGF2, and basement membrane extract (BME) to create an environment for angiogenesis. Being a mixture of extracellular matrix, BME can form an interface between epithelial, muscle, and stromal cells. It may provide a suitable microenvironment for angiogenesis. VEGF and FGF2, being potent angiogenic factors, are demonstrated to promote angiogenesis and graft survival [27,28,29]. Therefore, coupling of BME, VEGF, and FGF2 may promote revascularization of the ovarian implants during the critical time of transplantation. This could be the reason for the higher survival rate in this study.

In general, oxygen, nutrient, and gonadotropin supplements are essential for folliculogenesis of subcutaneously transplanted ovarian tissue. In the present study, we demonstrated that VEGF and FGF2 promoted revascularization of the ovarian graft; therefore, the survival rate of the graft was increased, and the size of the graft generally was larger than the untreated control graft, suggesting that ischemic injury can be improved to some extent and, thus, oxygen and nutrients could reach the graft. However, the ovarian response to gonadotropins seemed to be unexpected. Higher doses of gonadotropins were used because the general dose (e.g., 5 IU pregnant mare’s serum gonadotropin (PMSG) and 10 IU human chorionic gonadotropin (hCG)) routinely used in the normal mice could not well stimulate folliculogenesis of the ovarian grafts. The situation was also observed previously [30]. This phenomenon may reflect a poor ovarian reserve that affects the response to gonadotropin stimulation. While the two growth factors induced angiogenesis in ovarian grafts, and some larger vessels and more microvessels were detected, the coverage and the deep vessel may remain insufficient and impact the ovarian reserve. Another clue for poor ovarian reserve is the ratio of matured oocytes. Only an average of approximately 2.7 MII oocytes per grafted ovarian tissue can be retrieved from the VEGF/FGF2-treated graft. Consequently, poor ovarian response may be due to limited vascularization of the grafts in the subcutaneous space and poor ovarian reserve after freezing, thawing, and transplantation.

Despite untreated control with a relatively higher numbers of preantral and antral follicles without treatment with gonadotropins, the quality of oocytes seemingly damaged since a relatively low number of oocytes can be developed into the blastocyst stage [31]. Even after gonadotropin treatment, the quality of oocytes collected from the untreated group still is poorer as demonstrated by the very lower rate of blastocyst formation. In fact, the oocytes retrieved from subcutaneous transplanted ovaries showed a relatively lower fertilization rate compared with those normally-ovulated oocytes (Table S4).

The increases in the numbers of primordial and primary follicles were found by increasing doses of gonadotropin; however, these increases are not associated with gonadotropin treatment since follicles at these stages do not respond to FSH stimuli in nature. As taking the 150 IU group, with a larger sample size, for consideration, there are no differences in the number of primordial follicles, while the number of primary follicles is just reaching statistical significance (*p* = 0.0466) between the two groups. In addition, the numbers of primordial and primary follicles have no differences between the two groups without gonadotropins treatment (Table 1). Therefore, whether the numbers of primordial and primary follicles are increased in VEGF/FGF2-treated groups or increasing the sample size may change the differences needs further evaluation.

Counting sections of the grafted ovary found that the numbers of antral follicles in VEGF/FGF2-treated ovarian grafts were approximately three-fold higher than those in untreated controls. However, when oocytes were directly retrieved from antral follicles of the grafted ovaries, the differences of the retrieved oocytes between control and VEGF/FGF2 treatment may be reduced. The ovarian grafts were buried under the subcutaneous site of the inguinal region where the surface of the grafts were covered with a layer of tough connective tissues, it must be removed first during the oocyte retrieval, and only the larger antral follicles were collected. Nevertheless, many oocytes need to be picked up from a small grafted ovary; the scene is muddy and in a mess. Thus, some oocytes are possibly missing. In addition, we also need to consider the standard deviation found in different batches of experiment. This may explain why the differences of the retrieved oocytes between control and VEGF/FGF2 treatment were too small.

Despite the poor response of the ovarian grafts, more available MII oocytes were retrieved from the VEGF/FGF2-treated grafts compared with the control grafts. Ischemia may largely impair the ovarian reserve of the control grafts, leading to the only approximately 20% rate of MII oocyte recovery [31] as shown in our control group; however, oocyte quality is much better in VEGF/FGF2-treated ovarian than in the control grafts. More blastocyst embryos could be obtained under the treatment of VEGF and FGF2. Blastocyst has been reported to have better pregnancy and live birth rates in assisted reproductive technology [32,33], suggesting that taking the advantage of potent angiogenesis factors is beneficial for fertility preservation.

Protein levels of VEGF, FGF2, and some cytokines were relatively higher, but there was no statistical difference between the VEGF/FGF2-treated and untreated ovarian grafts one week after transplantation. This cannot be explained by inflammation after the graft operation. While inflammation can induce the expression of cytokines and growth factors, the operative procedure was the same between the VEGF/FGF2-treated and untreated control grafts. Nevertheless, the promotion of revascularization of the ovarian graft by VEGF/FGF2 must include biological processes such as wound healing and tissue remodeling. Thus, VEGF and FGF2 may induce the elevation of other angiogenic-related cytokines. The elevation disappeared two and three weeks after transplantation, indicating that the grafted ovary may recover to a stable status at least two weeks after transplantation as demonstrated by the similar levels of growth factors and cytokines between the two groups. The underlying causes regarding the increased levels of angiogenic cytokines were declined by longer cultivation time in bodies after grafting require further investigation.

The effect of growth factors on the survival and fertility potential of the ovarian graft is controversial. In monkeys, VEGF does not improve the viability of subcutaneously transplanted ovarian grafts [34]. Gao et al. used high concentrations of FGF2 (75–150 µg/mL) to treat the transplanted fresh ovarian tissue and demonstrated that FGF2 significantly improved primordial follicle survival and angiogenesis one week after transplantation, and apoptosis of follicles and stromal cells was significantly decreased [35]. However, they showed that lower dose of FGF2 (25 and 50 µg/mL) had no effect. In our study, combined VEGF (3 µg/mL) and FGF2 (9 µg/mL) showed beneficial effects on cryopreserved, subcutaneously transplanted ovarian tissues with a relatively low concentration of FGF2, even at eight- to 17-fold lower concentrations than used in their study. Recently, the authors mixed VEGF and FGF2 using a six- and three-fold higher concentration, respectively, compared with ours to treat subcutaneously transplanted cryopreserved ovarian tissues and demonstrated that the two growth factors can promote angiogenesis and enhance graft survival [36].

Kang et al. reported that culture of vitrified-thawed human ovarian tissues supplemented with a relatively low concentration of FGF2 (150 ng/mL) alone for two days and then subcutaneous xenografting to severe combined immune deficiency mice for one week significantly increased microvessel density and the number of follicles, while apoptosis significantly decreased. Their results showed that these effects were ascribed to FGF2 alone but not VEGF (25–100 ng/mL) [37]. A recent study reported that preinjection of VEGF into the subcutaneous site of Nu mice and then grafting cryopreserved ovaries increased the number of follicles at each stage [38]. These studies and ours all showed similar effects on cryopreserved, subcutaneously autotransplanted ovarian grafts with increases in blood vessels and survival rate. Unfortunately, the competence of oocytes retrieved from the ovarian grafts has not been assessed in the studies mentioned above. The novelty of this study is that we demonstrated that the quality of oocytes retrieved from subcutaneously transplanted mouse ovarian tissue has fertility potential. Nevertheless, whether these effects on the ovarian grafts are attributed to FGF2 alone or in combination with VEGF and what is the optimal concentration still needs to be determined.

In conclusion, VEGF and FGF2 promoted angiogenesis and significantly increased the survival rate of subcutaneously transplanted cryopreserved ovarian tissues. The number of various stages of follicles was prominently increased in the VEGF/FGF2-treated ovarian grafts after gonadotropin administration. In addition, oocyte quality was much better in the VEGF/FGF2-treated ovarian grafts. While cryopreservation of ovarian tissue followed by autotransplantation still is under investigation, combined VEGF and FGF2 might be the promising remedy to preserve fertility for children and young women with cancer.

## 4. Materials and Methods

### 4.1. Animals

Specific pathogen-free outbred CD-1 mice (BioLASCO Taiwan, Taipei, Taiwan) were bred in the Animal Center at the Department of Medical Research, Mackay Memorial Hospital following institutional guidelines for the care and use of experimental animals. The animal use protocol was reviewed and approved by the hospital’s Institutional Animal Care and Use Committee (approval number, MMH-A-S-97042). All efforts were made to minimize suffering. Animals were housed under controlled lighting (14 h light, 10 h dark) at 21–22 °C and were provided with water and chow ad libitum. Sexually mature (6–8-week old) female mice were used for the main study, while male mice (14-week old) were used for IVF.

### 4.2. Vitrification of Ovarian Tissues

The mice were injected intraperitoneally with 15 µL/g body weight of avertin solution, which was made by dissolving 1 g of 2,2,2-tribromoethanol (Sigma-Aldrich Chemical Co., St. Louis, MO, USA) in 1 mL of tertiary-amyl alcohol (J.T. Baker, Phillipsburg, NJ, USA) for mouse anesthesia. Both ovaries were removed, freed of fat, and cut into three equal pieces in M2 medium (Sigma-Aldrich) under a dissection microscope. Next, the six pieces of ovarian tissues were randomly mixed and immersed in MEMα medium (Life Technologies, Grand Island, NY, USA) supplemented with 12% fetal bovine serum (Sigma-Aldrich), 100 µg/mL streptomycin, and 100 µg/mL penicillin, pH 7.4, for 5 min. Afterward, the tissues were incubated in the same medium containing 2.0 mol/L dimethyl sulfoxide (DMSO) and 0.1 mol/L sucrose for 5 min, and then transferred into another fresh medium containing 2.0 mol/L DMSO, 0.2 mol/L sucrose, and 2.0 mol/L propanediol for 5 min. The tissues were vitrified by directly dipping in liquid nitrogen, transferred into the precooled cryovial, and stored in the liquid nitrogen tank.

### 4.3. Treatment with VEGF and FGF2, and Autologous Subcutaneous Transplantation of the Vitrified-Thawed Ovarian Tissue

A week later, cryovials were removed from liquid nitrogen, held in the air at room temperature for 20 s, and then the cryopreserved tissues were thawed quickly by immersing in 37 °C prewarmed phosphate-buffered saline (PBS). Once thawed, the tissues were consecutively diluted in MEMα medium containing 0.5, 0.25, and 0.125 mol/L sucrose, respectively, for 5 min each. After briefly rinsing in MEMα medium three times, the tissues were cultured in M2 medium in a humidified 5% CO_2_ atmosphere at 37 °C for 15 min. Simultaneously, VEGF and FGF2 were mixed with BME and heparin solution to prepare a final concentration of 3 and 9 µg/mL, respectively, on ice to prevent gel formation according to the instructions in the Directed In Vivo Angiogenesis Assay starter kit (Trevigen, Gaithersburg, MD, USA). Subsequently, the ovarian pieces wiped with a tissue paper were soaked in 10 µL premix reagent, with or without VEGF/FGF2, at 37 °C on a rack provided by the kit to form a gel-like substance, and then transplanted in the inguinal region of the mouse.

After the mouse was anesthetized with avertin, an approximately 0.3 cm incision was made in the inguinal region, followed by blunt dissection of a 1-cm subcutaneous pocket using a pair of fine curved watchmaker’s forceps. Three ovarian pieces treated with VEGF, FGF2, and BME were inserted into one side of the subcutaneous space, and the other three pieces mixed with BME, but without VEGF and FGF2, were inserted into the opposite side of the same mouse. Finally, the skin incision was closed with a single nylon suture.

### 4.4. Graft Retrieval and Histological Analysis of Follicles

Ovarian grafts were retrieved two and three weeks after transplantation for analysis of morphology and graft survival. The survival rate was defined as the percentage of ovarian grafts still present at the retrieval time relative to the total ovarian grafts. The untreated control group or the VEGF/FGF2-treated group was counted independently. Only those grafts where all survived from the control and VEGF/FGF2-treated groups in the same mouse were used for the following assay. The retrieved grafts, which had been grafted for two weeks, were fixed in neutral formalin, embedded in paraffin, and cut into 5-μm sections for morphology observation or immunohistochemical staining. Mice grafted with ovarian tissue three weeks after transplantation were treated with gonadotropins. Three dosages of pregnant mare’s serum gonadotropin (PMSG, Sigma-Aldrich), i.e., 50, 150, and 250 international units (IU), were injected intraperitoneally, and an equivalent dose of human chorionic gonadotropin (hCG, China Chemical and Pharmaceutical, Hsinchu, Taiwan) was administered 48 h later. The mice were killed by cervical dislocation 16 h after hCG administration. The ovarian grafts were retrieved, fixed, and embedded in paraffin as aforementioned. Ten serial sections were stained with hematoxylin and eosin (H&E). Follicles were counted via light microscopy at three randomly selected fields in 10 serial sections. Follicles were classified as described previously [39].

### 4.5. Immunohistochemical Staining of Vessels

We stained for von Willebrand factor (vWF), one of the markers for the identification of endothelial cells of blood vessels, to recognize the blood vessel. After the ovarian sections on slides were deparaffinized and hydrated, tissues were demasked with antigen retrieval AR-10 solution (BioGenex, San Ramon, CA, USA); the slides were treated with 3% (*v*/*v*) H_2_O_2_ in PBS for 15 min, blocked with 10% (*v*/*v*) normal goat serum in PBS (blocking solution) for 1 h at room temperature, and then incubated with rabbit anti-vWF antiserum (ab6994; Abcam, Cambridge, UK) or the control antiserum diluted 1:1000 in blocking solution at 4 °C for 16 h. After washing, the slides were treated with biotin-conjugated goat anti-rabbit IgG (3 μg/mL; Zymed Laboratories, South San Francisco, CA, USA) for 1 h at room temperature. After washing, slides were incubated with 1 μg/mL of horseradish peroxidase (HRP)-conjugated streptavidin (Zymed Laboratories) for 40 min at room temperature. The slides were immunostained by 3-amino-9-ethylcarbazole staining (Zymed Laboratories), counterstained with hematoxylin (Vector Laboratories, Burlingame, CA, USA), and photographed using an Olympus BX 40 microscope (Olympus, Tokyo, Japan) equipped with an Olympus DP-70 digital camera.

### 4.6. Enzyme-Linked Immunosorbent Assay (ELISA)

Mouse angiogenesis ELISA strips for profiling eight cytokines (EA-1021; Signosis, Santa Clara, CA, USA), including VEGF and FGF2, were applied to determine the relative levels of angiogenic factors in the grafted ovarian tissues. Ovarian grafts were removed one, two, or three weeks after transplantation and were homogenized in PBS containing the protease inhibitor cocktail to prepare the total protein extract. The protein concentration was determined using a bicinchoninic acid protein assay kit (Pierce, Rockford, IL, USA). The protein extract (30 µg) was subjected to analysis according to the manufacturer’s instructions.

### 4.7. Gonadotropin Administration and Oocyte Retrieval

We superovulated the transplanted mice three weeks after grafting. Approximately 150 IU of PMSG was injected intraperitoneally, and an equivalent dose of hCG was given 48 h later. The transplanted ovarian tissues were removed carefully 16 h after hCG administration from the inguinal areas, and oocyte-granulosa cell complexes from the antral follicles were isolated using 30-gauge needles (Becton Dickinson, Bedford, MA, USA). After washing with M16 medium, the maturation status of the oocytes was scored as a germinal vesicle (GV) being present as an enlarged nucleus, a metaphase I (MI) referring to the breakdown of the GV, and a metaphase II (MII) when the first polar body had been extruded.

### 4.8. In Vitro Fertilization (IVF) and Embryo Culture

Mature MII oocytes retrieved from the transplanted ovarian tissues were subjected to IVF following the method reported previously [40]. The zygotes were cultured in M16 medium in a humidified 5% CO_2_ atmosphere at 37 °C for four days.

### 4.9. Statistical Analysis

Data are presented as the mean ± SD. The differences were analyzed by a paired Student’s *t*-test using GraphPad Prism 5 (San Diego, CA, USA). *p* < 0.05 was considered significant.

## Figures and Tables

**Table 1:** Comparison of the average number of follicles in subcutaneously transplanted cryopreserved ovarian tissue, treated with or without vascular endothelial growth factor (VEGF) and fibroblast growth factor 2 (FGF2), after gonadotropin administration.

Gonadotropins	Untreated Control				VEGF/FGF2 Treatment			
IU	PMF	PF	PAF	AF	PMF	PF	PAF	AF
0	22.50 ± 10.65	17.33 ± 6.12	12.00 ± 2.97 **	12.00 ± 4.69	21.17 ± 21.08	13.67 ± 11.06	6.33 ± 3.88	6.33 ± 6.71
50	22.14 ± 14.37	12.29 ± 10.72	5.86 ± 7.31	6.71 ± 5.71	38.43 ± 17.60 *	27.71 ± 13.78 *	8.14 ± 7.38	13.14 ± 10.56
150	29.36 ± 13.50	13.91 ± 10.89	8.64 ± 5.92	6.73 ± 6.07	36.64 ± 22.35	24.09 ± 13.74 *	11.73 ± 6.48	17.91 ± 12.19 *
250	15.75 ± 12.09	9.75 ± 8.66	4.25 ± 3.86	4.50 ± 5.26	52.75 ± 13.84 **	39.25 ± 15.11 *	18.00 ± 11.80	18.00 ± 16.75

Data represent the mean ± SD of the number of follicles at each stages (for 0, 50, 150, and 250 IU, *n* = 6, 7, 11, and 4, respectively). * *p* < 0.05, ** *p* < 0.01 compared with the untreated control. IU, international unit; PMF, primordial follicle; PF, primary follicle; PAF, preantral follicle; AF, antral follicle.

**Table 2:** Outcomes of oocyte maturation in antral follicles collected from cryopreserved, subcutaneously-transplanted ovarian tissue, treated with or without VEGF and FGF2, after gonadotropin administration.

Oocyte Status	No. Oocytes—Control	No. Oocytes—VEGF/FGF2
GV	111 (69%)	98 (51%)
MI	18 (11%)	28 (14%)
MII	33 (20%)	65 (35%)
Total No. oocytes	162	191

Three weeks after transplantation, mice (*n* = 24) grafted with ovarian tissue were treated with 150 IU gonadotropins, and various stages of oocytes were collected from the antral follicles of the ovarian grafts. GV, germinal vesicle; MI, metaphase I; MII, metaphase II.

## Supplementary Materials

Supplementary materials can be found at http://www.mdpi.com/1422-0067/17/8/1237/s1.
