# Supplementary material for: VEGF and FGF2 Improve Revascularization, Survival, and Oocyte Quality of Cryopreserved, Subcutaneously-Transplanted Mouse Ovarian Tissues
Source: Int J Mol Sci. 2016 Jul 30;17(8):1237. doi: 10.3390/ijms17081237 (PMC5000635; doi:10.3390/ijms17081237)
Supplement: Supplementary file 1 [file ijms-17-01237-s001.pdf]

Supplementary Materials: VEGF and FGF2 Improve Revascularization, Survival, and Oocyte Quality of Cryopreserved, Subcutaneously-Transplanted Mouse Ovarian Tissues

Sheng-Hsiang Li, Yuh-Ming Hwu, Chung-Hao Lu, Hsiao-Ho Chang, Cheng-En Hsieh and Robert Kuo-Kuang Lee

Table S1. Status of the grafted ovarian tissue, treated with or without vascular endothelial growth factor (VEGF) and fibroblast growth factor 2 (FGF2), two and three weeks after subcutaneous transplantation.

Tissue Status	Control No. (%)	VEGF/FGF2 No. (%)
2 W (n = 32)		
fibrosis	11 (34%)	2 (6%)
survival	21 (66%)	30 (94%)
3 W (n = 28)		
fibrosis	14 (50%)	5 (18%)
survival	14 (50%)	23 (82%)

The cryopreserved mouse ovarian tissues were autologously transplanted to the subcutaneous site of the inguinal region. Half of the ovarian tissues treated with VEGF/FGF2 were implanted into one side of the subcutaneous space, and the other half ones without treatment of VEGF/FGF2 were inserted into the opposite side of the same mouse. Two or three weeks after transplantation, ovarian grafts were retrieved for analysis of the tissue status. The fibrosis or survival rate was defined as the percentage of ovarian grafts absorbed or present at the retrieval time relative to the total ovarian grafts. The untreated control group or the VEGF/FGF2-treated group was counted independently.

Table S2. The numbers of oocytes retrieved from antral follicles of the transplanted ovarian tissues, treated with or without VEGF and FGF2, three weeks after subcutaneous transplantation.

**Experimental No.**	**Control**	**VEGF/FGF2**
1st exp. (n = 4)		
GV	23	12
MI	3	4
MII	6	15
2nd exp. (n = 4)		
GV	19	14
MI	2	7
MII	6	7
3rd exp. (n = 4)		
GV	15	18
MI	2	6
MII	7	9
4th exp. (n = 4)		
GV	22	10
MI	5	5
MII	4	12
5th exp. (n = 4)		
GV	15	20
MI	4	4
MII	4	10
6th exp. (n = 4)		
GV	17	24
MI	2	2
MII	6	12

Three weeks after transplantation, mice (n = 24) grafted with ovarian tissue were treated with 150 IU gonadotropins. Oocytes were retrieved from antral follicles of the ovarian grafts. Six independent experiments were performed. GV, germinal vesicle; MI, metaphase I; MII, metaphase II.

Table S3. Raw data for calculating the rate of MII oocytes, fertilization rate, and blastocyst formation rate.

Experimental No.	Control No. (%)	VEGF/FGF2 No. (%)
MII Rate [a]		
1	6 (19%)	15 (48%)
2	6 (22%)	7 (25%)
3	7 (29%)	9 (27%)
4	4 (13%)	12 (44%)
5	4 (17%)	10 (29%)
6	6 (24%)	12 (32%)
Fertilization Rate [b]		
1	3 (50%)	8 (53%)
2	2 (33%)	2 (29%)
3	4 (57%)	5 (56%)
4	2 (50%)	6 (50%)
5	3 (75%)	4 (40%)
6	3 (50%)	5 (42%)
Blastocyst Rate [c]		
1	0 (0%)	2 (25%)
2	0 (0%)	1 (50%)
3	1 (25%)	1 (20%)
4	0 (0%)	2 (33%)
5	1 (33%)	1 (25%)
6	0 (0%)	1 (20%)

[a] The number of MII oocytes and the percentage relative to all oocytes retrieved from the antral follicles; the data are extracted from the Table S2; [b] The number of fertilized MII oocytes and the percentage relative to all MII oocytes; [c] The number of blastocyst developed from the fertilized MII oocytes and the percentage relative to all fertilized oocytes.

Table S4. Outcomes of in vitro fertilization (IVF) using normally-ovulated oocytes.

Experimental No.	Oocyte No.	Fertilized Oocyte No.	Fertilization Rate [a] (%)
1	33	22	67
2	24	15	63
3	15	12	80
4	17	13	76
5	27	17	63

[a] Fertilization rates were expressed as the percentage of two-cell embryos relative to the total oocytes at 24 h after IVF.
